# The applicability of effective connectivity measures to time series of neuronal oscillators

**DOI:** 10.1186/1471-2202-12-S1-P324

**Published:** 2011-07-18

**Authors:** Erin R Boykin, William O Ogle, Paul R Carney, Pramod P Khargonekar, Sachin S Talathi

**Affiliations:** 1 Department of Electrical and Computer Engineering, University of Florida, Gainesville, FL 32611, USA; 2 Department of Biomedical Engineering, University of Florida, Gainesville, FL 32611, USA; 3 Department of Pediatrics, University of Florida, Gainesville, FL 32611, USA; 4 Department of Pediatrics, Neuroscience, and Biomedical Engineering, University of Florida, Gainesville, FL 32611, USA

## 

The use of time series analysis techniques such as Granger causality (GC), partial directed coherence (PDC) and phase dynamics modeling (PDM) to estimate effective connectivity in brain networks has recently gained significant prominence in the neuroscience community. While these techniques have been useful in determining the effective connectivity structure of brain networks [1,2], the results they produce can be incorrect when the recording channels have a high degree of coherence [3] or a large bias in their degree of noise [4]. Nevertheless, it is standard practice to apply these techniques to multichannel datasets with little or no regard for whether the channels fall into one of these problematic categories. In this work, we address this issue within the context of simple networks of coupled spiking neurons. Specifically, we develop a method based on decision tree classifiers to assess the ability of GC, PDC, generalized PDC, and PDM to accurately determine effective connectivity in a neuronal network.

We begin by generating an ensemble of time series datasets by systematically varying the parameters of a simple network of two coupled Morris-Lecar (ML) neurons. The varied parameters include the coupling type (linear diffusive, nonlinear fast threshold, nonlinear synaptic), coupling strength, intrinsic firing rates and noise levels. Next, we characterize each dataset of the ensemble using the following time series features: i) phase coherence, ii) difference in the coefficient of variation of interspike intervals, iii) mean coefficient of variation of interspike intervals, and iv) difference in firing frequency. We then apply four effective connectivity measures to the datasets in the ensemble to obtain estimates for the direction of interactions in the network. These results are used to label the datasets as “correct” (+1) or “incorrect” (0) depending on whether the measure correctly or incorrectly predicts the known connectivity structure of the underlying ML network. Finally, we use the calculated time series features and the labeled ML network datasets to develop a decision tree classifier.

We apply the decision tree classifiers to larger networks of three neuronal oscillators by considering time series features for all pairwise interactions in the network and determining the conditions under which the effective connectivity measures can successfully detect directionality of coupling in these systems. We find that if a majority of pairwise interactions in the network are classified as 0 for a given measure, then that measure should not be applied since it will most likely (probability >50%) result in failure. Extending our observations to multichannel datasets with greater than three nodes, we conclude that if all pairwise interactions in a network are classified as 0 by our decision tree classifier, then a given measure should not be applied to the data since effective connectivity estimates are likely to be incorrect.

## References

[B1] Cadotte AJ DeMarse TB Mareci TH Parekh MB Talathi SS Hwng DU Ditto WL Ding M Carney PR Granger causality relationships between local field potentials in an animal model of temporal lobe epilepsy J Neurosci Methods 2010 189 121 129 20304005 10.1016/j.jneumeth.2010.03.007 PMC2867107

[B2] Sato JR Takahashi DY Arcuri SM Sameshima K Morettin PA Baccala LA Frequency domain connectivity identification: An application of partial directed coherence in fMRI Human Brain Mapping 2009 30 452 461 10.1002/hbm.20513 18064582 PMC6871135

[B3] Smirnov D Schelter B Winterhalder M Timmer J Revealing direction of coupling between neuronal oscillators from time series: Phase dynamics modeling versus partial directed coherence Chaos 2007 17 013111 10.1063/1.2430639 17411247

[B4] Nalatore H Ding M Rangarajan G Mitigating the effects of measurement noise on Granger causality Phys Rev E 2007 75 031123 10.1103/PhysRevE.75.031123 17500684

